# Women's relative immunity to the socio-economic health gradient: artifact or real?

**DOI:** 10.3402/gha.v8.27259

**Published:** 2015-05-05

**Authors:** Susan P. Phillips, Katarina Hamberg

**Affiliations:** 1 Departments of Family Medicine and Public Health Sciences, Queen's University, Kingston, ON, Canada; 2 Umeå Centre for Gender Studies, Umeå University, Umeå, Sweden; 3 Division of Family Medicine, Department of Public Health and Clinical Medicine, Umeå University, Umeå, Sweden

**Keywords:** gender, women, men, socio-economic status, sex factors, mortality, morbidity, cardiovascular disease, social capital, deprivation index

## Abstract

**Background:**

Individual and area socio-economic status (SES) are significant predictors of morbidity and mortality in developed and developing countries. However, the span in health from poorest to richest, that is, the socio-economic gradient, appears steeper for men than women.

**Objective:**

Our aim is to understand women's apparent immunity to the health harms of the SES gradient.

**Design:**

Findings from a non-systematic search of Medline for population-based, SES gradient studies reporting results for both men and women and with health outcomes of morbidity, mortality or self-rated health (SRH) were reflectively analyzed.

**Results:**

The 36 papers reviewed generally showed women to be relatively immune to the SES gradient for all but cardiovascular health outcomes. However, addressing the interconnected nature of socio-economic circumstances, exploring whether some measures of SES had ambiguous meanings for either women or men, including modifiers of SES such as household circumstances, social capital or area gender equity, or using indicators of area SES that were contextual rather than aggregates of individual, compositional measures increased the SES gradient for women. Outcome measures that combined mental and physical health, accounted for gender differences in SRH and adjusted for sex-specific differences in causes of mortality also explained some of the observed amelioration of the SES gradient among women.

**Conclusions:**

Socio-economic circumstances have a real and sustained impact on individual health. The SES gradient appears stronger for men than for women for all health outcomes other than heart disease. However, some of the observed variability between men and women may be an artifact of biased methodology. Considering webs of causation rather than individual markers of SES along with other sources of gender bias can explain much of women's blunted socio-economic gradient and deepen understanding of the pathways from SES to morbidity and mortality overall.

Research from many countries and across decades documents the connection between material deprivation and excess morbidity and mortality (1). This socio-economic status (SES) gradient in health extends from the bottom to the top of the SES spectrum with increments in individual SES, however measured, conferring incremental health benefits. The SES of neighborhoods appears to have a similar and independent, although weaker place effect on the health of all who live within their boundaries (2). Poor people living in poor neighborhoods are, therefore, particularly disadvantaged.

There are, however, some disquieting gender-related inconsistencies in the evidence. These do not negate findings that social and economic inequities adversely affect health, or the moral and ethical obligation to protect those who are disadvantaged. However, the source of inequity may not be as singular as socio-economic position. What are the gendered nuances of the evidence and what might they mean? This is the subject of the reflective analysis that follows.

## Are women insulated from the SES gradient?

When data for men and women are disaggregated, the gradient from socio-economic circumstances to all-cause mortality generally appears blunted for women relative to men, whether SES measures used are individual, ecological, or multilevel (3). At the same time, women outlive men throughout the world. Despite their relative poverty, deprivation, and constrained access to resources, the impact of the socio-economic gradient on women appears to be dampened, with life expectancy exceeding men's at each level of SES and a narrower span between the health of poorest and richest (4). There are few conceptual explanations for the apparent resistance of women's health to socio-economic deprivation or advantage. These gender differences raise questions as to the meanings of and explanations for associations between SES and illness, overall, and gender-related variations in this relationship.

## Present investigation

Is gender intertwined in the pathway from SES to health? Our aim is to explore whether observed differences in men's and women's susceptibility to socio-economic harm or benefit endure when gender biases in the research questions asked, indicators used, and interpretations of findings are minimized. This exploration may add clarity to definitions of social determinants of health, in general, and identify which social circumstances enter the body to shape biology. We will review cumulative and current evidence of the impact on women's and men's health of material circumstances. Is this evidence consistent and trustworthy or might it be illusory, arising from flawed methodology or choice of indicators with embedded gender bias? Are there missing modifiers of the association between SES and health that explain observed effect differences between women and men? Do sex specific differences in causes of death distort the apparent SES gradient?

### Women and men: individuals or groups?

Morbidity and mortality are not distributed evenly or randomly in any population. While innate biology does confer risk, vulnerability also arises from the ‘baggage’ of social circumstances or deprivations concomitant with membership in a given group. The biological categories of ‘men’ and ‘women’ are generally fixed and lack the fluidity typical of groups studied in cluster analyses. Nevertheless, there may well be gendered characteristics imposed upon, or similar among all women or all men in a particular society. Deciding whether to consider all those being studied as individuals or, instead, to check if group membership (with groups ‘men’ or ‘women’) has a ‘cluster’ effect is both a philosophical and a methodological issue. If one believes that the similarities inherent in being human override variability arising from group traits, then those group effects will not be of substance or relevance. On the other hand, a fundamental assumption of statistical methodology is the independence of those in a sample. If there are group-level characteristics arising from being, for example, a woman, then these must be examined along with looking for individual variation within groups. This methodological conundrum underlies our exploration.

### SES and health indicators

Throughout this paper, the terms SES and socio-economic position will be used interchangeably. Measures of individual or household SES used in health outcomes research are generally single indicators of individual status such as income, adequate access to resources, or education. Area SES may be quantified via individual characteristics aggregated up to a neighborhood or ecological level (e.g. average area education), by developing area measures that combine several neighborhood characteristics into a single index as do the Townsend and Carstairs indices (5, 6), or by using an upstream indicator with no individual-level correlate or meaning (e.g. crime rates or local access to resources). Typical outcomes studied include overall or cause-specific mortality, physical illness, self-rated health (SRH), or prevalence of risk factors for disease such as smoking or obesity.

### Sex, gender, and health

Health differences between women and men cannot be reduced to sex, that is, to genetic or other biological factors. Instead, the term gender more appropriately acknowledges that experiences, behavior, health, and disease are embedded in an interconnected web of political, cultural, psychological, and socio-economic conditions and that being a woman or a man implies differences in part or all of that web of causation (7). Gender is about populations and their collective lives as well as individual bodies. Although genetics may predispose individuals or groups to illness or premature mortality, environment and life experiences flip the epigenetic switches, turning on or off those inherent genetic risks of disease. The interaction between genetics, biological processes, expectations and norms for what it means to be a man or a woman, and lived experiences is what we think of as sex/gender but will refer to, in this paper, simply as gender. In other words, conceptually, gender will be used here to combine biological sex with social attributes and constraints afforded by or demanded differentially of men and women in a specific context, group, culture, or country.

## Methods

### The search for sex-stratified SES gradient studies

To grasp the breadth and depth of social gradient research, we began by searching for SES gradient studies that reported results for women and men separately. Finding such research is challenging because medical subject headings (MeSH) lack clarity regarding the terms sex, gender, and equity (8). This is a literature that cannot be reined in despite repeated searches of keywords, titles, and abstracts using various terms, and relevant papers were likely missed. Starting with a Medline (National Library of Medicine) search of the keywords women, men, female, male, socio-economic factors, health status indicators, and sex factors yielded such a jumble of papers that we refined our strategy. Our next strategy involved merging results of three separate keyword searches: 1) adult, education, marital status, mortality, occupation, and socio-economic factors; 2) adult, coronary disease, educational status, women, men, human, sex distribution, and social class; 3) adult, educational status, women, men, human, sex distribution, social class, and mortality. Among outcome indicators of morbidity, we specifically included coronary artery disease as it is equally common in men and women and, in the developed world, is the leading cause of mortality for all. In a less systematic manner, we then identified ‘related papers and citations’ of relevance. Articles that addressed only one sex, failed to report findings in a manner that allowed for comparison of women and men, duplicated data reported in another included study, were primarily time-series analyses, were not population-based, or limited outcomes to mental health or an intermediate risk factor such as obesity or smoking were excluded, while review articles were included. This left 36 papers to guide our reflective analysis of gender differences in the SES gradient and what they mean (9–44). These papers are listed among the references, summarized in Table 1 and cited in the remainder of this paper. Each paper was read and reread to identify SES and health outcome measures, level of analysis (individual, ecological, or multilevel), whether and how area gender equality was included, and how sex/gender was addressed in methodology and outcomes. The first author then developed a list of concepts and themes arising that both authors discussed, debated, and refined. These themes are reflected in the subheadings of the next section.

**Table 1:** Summary table of reviewed papers (ordered by author)

First author. Title. Year (Reference number)	Setting, study population	Level of analysis: Individual=i Ecological=e Multilevel=m	Measures of social status	Health outcome measure	How sex/gender addressed in methodology	SES gradient greater for men than women
Backhans. Does increased gender equality lead to a convergence of health outcomes for men and women? A study of Swedish municipalities. 2007 (9)	Sweden Whole population 2000–2004	e	Gender equality measured as political participation Division of labor, employment proportions in typically segregated sectors, income ratios	Life expectancy Sick days	Sex-disaggregated linear regression including independent variables of gender equality	Yes
Bopp. Mortality by education in German speaking Switzerland, 1990–1997: results from the Swiss National Cohort. 2003 (10)	Switzerland Approximately 75% of German–Swiss population Age 25+ 1990–1997	i	Education	Mortality	Sex-disaggregated mortality ratios and regressions	Yes
Borrell. Social class and self-reported health status among men and women: what is the role of work organization, household material standards and household labour? 2004 (11)	Barcelona *N*=2,345 (m), 1,874 (w) Age 16–64 2000	i	Occupation, social class, psychological and physical working conditions, job insecurity, hours worked, home amenities, household labor	Self-rated health	Sex-disaggregated logistic regression	Yes for all measures of SES except household labor
Deguen. A small-area ecologic study of myocardial infarction, neighborhood deprivation, and sex. 2010 (12)	Strasbourg *N*=1,193 Age 35–74	e	Neighborhood deprivation index (income, education, job, housing, family structure, immigrants)	Myocardial infarction	Interaction terms of sex^x^ deprivation index	No, SES gradient greater for women
Drever. Exploring the relation between class, gender, and self rated general health using the new socioeconomic classification. A study using data from the 2001 census. 2004 (13)	Britain *N*=30.3 million Age 25–64	i	Multiple aspects of employment	Self-rated health	Sex-disaggregated rates of levels of SRH for levels of socio-economic position	Yes
Eriksson. The importance of gender and conceptualization for understanding the association between collective social capital and health: a multilevel analysis from northern Sweden. 2011 (14)	Sweden *N*=3,225 (w) 2,543 (m). Age 18–84	m	Neighborhood social capital Individual social capital Individual socio-demographics: age, education, income, marital status, children at home, country of birth	Self-rated health	Sex-disaggregated multilevel analyses	No, SES gradient greater for women
Ferrie. Self-reported job insecurity and health in the Whitehall II study: potential explanations of the relationship. 2005 (15)	Britain *N*=2,145 (w), 5,052 (m)	i	Job security Education, marital status, material deprivation, psychological status, job satisfaction and control, alcohol, smoking	Self-rated health Long-standing illness Minor psychiatric morbidity	Sex-disaggregated regression analyses	No, SES gradient greater for women
Huisman. Educational inequalities in cause-specific mortality in middle-aged and older men and women in eight western European populations. 2005 (16)	Europe 51 million person years, all deaths 1990–1997	i	Education level	Cause-specific mortality	Sex-disaggregated regression	Yes
Kawachi. Women's status and the health of women and men: a view from the States. 1999 (17)	US Census population 1990s	e	Gender equality index (women's political participation, economic autonomy, employment/earnings, reproductive rights) income inequality, poverty rate, median household income	Mortality	Sex-disaggregated regression analyses	Yes for all indicators except reproductive rights
Kelleher. Socio-demographic predictors of self-rated health in the Republic of Ireland: findings from the National Survey on Lifestyle, Attitudes and Nutrition, SLAN. 2003 (18)	Ireland *N*=6,539	m	Neighborhood deprivation index Individual data: age, marital status, education, household occupation class, household size, marital status, housing, rurality, smoking, disease diagnosis	Self-rated health	Sex-disaggregated multilevel analyses	Yes
Kopp. Low economic status of the opposite sex is a risk factor for middle aged mortality. 2005 (19)	Hungary Population sample by 150 regions Age 18+	e	Subjective social position (women), average education, average income	Mortality	Sex-disaggregated regression analyses	Yes
Koskinen. Why are socioeconomic mortality differences smaller among women than men? 1994 (20)	Finland, census 1980 Age 35–64	i	Education, occupation, housing density, dwelling standard, marital status, area of residence	Cause-specific mortality	Sex-disaggregated mortality differences, interactions	Yes
Ljung. Socioeconomic differences in the burden of disease in Sweden. 2005 (21)	Sweden Whole population Age 15–84	i	Occupation, Disease	DALYs	Sex-disaggregated regression	Yes
Mackenbach. Socioeconomic inequalities in mortality among women and among men: an international study. 1999 (22)	US, Finland, Norway, Italy Czech Republic, Hungary, Estonia 1980–1990 N – not reported	i	Education, age, race/ethnicity (for US only)	Mortality	Sex-disaggregated regression analyses	Yes for all outcomes except cardiovascular diseasesome variation by country
Mackenbach. Socioeconomic inequalities in health in 22 European countries. 2008 (23)	Europe, whole population Age 30–69 1990–2000	i	Education, occupation, income age, self-rated health, smoking, obesity	Cause-specific mortality	Sex-disaggregated data	Yes for all outcomes except cardiovascular mortality
Major. Neighborhood socioeconomic deprivation and mortality: NIH-AARP diet and health study. 2010 (24)	US *N*=556,402, (33,831 deaths) Age 50–80 1995–2005	m	Neighborhood deprivation index Individual data: dietary intake, activity, medical history, BMI	Mortality	Sex-disaggregated regressions and multi-level analyses	Yes
Malyutina. Education, marital status, and total and cardiovascular mortality in Novosibirsk, Russia: a prospective cohort study. 2004 (25)	Russia Random sample *N*=6,485 (m) 4,919 (w) Age 25–64 in 1984	i	Education, marital status, age, smoking, blood pressure, BMI, alcohol, cholesterol	All-cause mortality	Sex-disaggregated regressions	No, SES gradient greater for women but non-linear
Martikainen. Income differences in mortality: a register-based follow-up study of three million men and women. 2001 (26)	Finland *N*=261,000 deaths Age>30 1991–1996	i	Household income Household employment, marital status, education, economic activity, spouse's SES	Mortality	Sex-disaggregated regressions	Yes
Meijer. Do neighborhoods affect individual mortality? A systematic review and meta-analysis of multilevel studies. 2012 (27)	Systematic review, developed countries Before 2010	m	Area indicators of social cohesion, income inequality, social capital Individual SES measures	Mortality	Unclear – controlled for sex in some analyses	Yes
Muntaner. The associations of social class and social stratification with patterns of general and mental health in a Spanish population. 2003 (28)	Barcelona *N*=4,218 Age 16–64 2000	i	Ownership and control of productive assets, social stratification, education	Self-rated health Mental health (GHQ)	Sex-disaggregated regression analyses	Yes but non-linear and limited significance for either
Naess. Childhood and adulthood socioeconomic position across 20 causes of death: a prospective cohort study of 800,000 Norwegian men and women. 2007 (29)	Norway *N*=79,534 Age 0–20 in 1960 1990–2001	i	Parents’ occupation, household income	Cause-specific mortality	Sex-disaggregated regressions	Yes
Nicholson. Socio-economic influences on SRH in Russian men and women – a life course approach. 2005 (30)	Russia Random sample *N*=1,004 (m), 1,930 (w) Age>50 2002	i	Childhood adversity, education, perceived class, household income, marital status, alcohol consumption, smoking	Self-rated health	Sex-disaggregated regression analyses	Yes
Perel. Household wealth and the metabolic syndrome in the Whitehall II Study. 2006 (31)	Britain *N*=1,509 (w), 4,090 (m) Age 45–68	i	Household income, own income household wealth, marital status, education level, father's occupation, household size	Metabolic syndrome	Sex-disaggregated regression analyses	No, SES gradient greater for women
Phillips. Relative health effects of education, socioeconomic status and domestic gender inequity in Sweden: a cohort study. 2011 (32)	Sweden *N*=773 Age=42 2007	i	Education financial strain domestic equality	Self-rated health	Sex-disaggregated regression analyses	Yes for domestic equality, but reversed for financial strain and education
Rey. Ecological association between a deprivation index and mortality in France over the period 1997–2001: variations with spatial scale, degree of urbanicity, age, gender and cause of death. 2009 (33)	France Census population 1997–2001	e	Neighborhood deprivation index, urbanicity, Townsend index, Carstairs index	Mortality	Sex-disaggregated mortality differentials	Yes
Roberts. Macro-level gender equality and alcohol consumption: a multilevel analysis across U.S. States. 2012 (34)	US *N*=200,000 2005	m	Area gender equality indices×5 (women's SES, gender equality in SES, political participation, reproductive rights, violence policies) Area: income and income inequality, median income, religion, Individual: age, race, income, marital status, education, employment	Alcohol consumption	Sex-disaggregated multilevel and multiple analyses	Greater equity associated with decreased drinking for men and women
Sacker. Comparing health inequality in men and women: prospective study of mortality 1986–96. 2000 (35)	Britain *N*=235,083 Age 16–65 (m), 16–60 (w)	i	Household social position, occupation	Mortality	Sex-disaggregated regression analyses	Yes for occupation but women's SES gradient exceeds men's for household position
Saurel-Cubizolles. Social inequalities in mortality by cause among men and women in France. 2009 (36)	France *N*=104,109 (men), 109,765 (women) Age 30–64 (as of 1990)	i	Education, occupation	All-cause mortality Cancer mortality Injury mortality Cardiovascular mortality	Sex-disaggregated data	Yes except for cardiovascular mortality where women's SES gradient exceeds men's
Seubsman. Gender, socioeconomic status, and self-rated health in a transitional middle-income setting: evidence from Thailand. 2011 (37)	Thailand *N*=87,134 Median Age=29 2005	i	Education, individual income, household assets, occupation marital status, urbanicity	Self-rated health	Sex-disaggregated regression analyses	Yes
Singh. Area, deprivation and widening inequalities in US mortality, 1969–1998. 2003 (38)	US Census population 1969–1998	e	Deprivation index	Mortality	Sex-disaggregated, multiple methods	Yes
Smith. Individual social class, area-based deprivation, cardiovascular disease risk factors, and mortality: the Renfrew and Paisley study. 1998 (39)	Scotland *N*=6,961 (men), 7,991 (women) Age 25–64 in 1972	m	Carstairs deprivation index (1972) Individual or husband's occupation (1972) Measures of personal health	CVD mortality All-cause mortality	Sex-disaggregated, multiple methods	Yes
Steenland. All-cause and cause-specific mortality by socioeconomic status among employed persons in 27 US states, 1984–1997. 2004 (40)	US Age 35–64 1984–1997	i	Occupation	Cause-specific mortality	Sex-disaggregated regression analyses	Yes
Stjarne. Socioeconomic context in area of living and risk of myocardial infarction: results from Stockholm Heart Epidemiology Program (SHEEP). 2002 (41)	Sweden *N*=Stockholm population Age 45–70 1992–1994	m	Neighborhood deprivation index Individual data: occupation, education, employment, marital status, birth country	Myocardial infarction	Sex-stratified multilevel regressions	No, women's SES gradient exceeds men's
Stringhini. Socioeconomic status, structural and functional measures of	Britain, Whitehall II *N*=6,895 (men), 3,413	i	Social support, Marital status	Mortality	Sex-disaggregated regression analyses	Yes
social support, and mortality. 2012 (42)	(women) Age 35–55 in 1985–1988	i	Social connectedness Occupation 1985–1988	Mortality	Sex-disaggregated regression analyses	Yes
Thurston. Is the association between socioeconomic position and coronary heart disease stronger in women than men? 2005 (43)	US NHANEs, *N*=2,750 (men), 3,275 (women) Age 25–74 (in 1971–1974)	i	Education, household income, single parent, employment, depression, smoker, alcohol use, physical activity, hypertension, diabetes, BMI, cholesterol, race	Coronary heart disease	Sex-disaggregation of multiple methods	No, women's SES gradient exceeds men's
Thurston. Women, loneliness, and incident coronary heart disease. 2009 (44)	US NHANEs, *N*=1,150 (men), 1,466 (women) Age 25–74 (in 1971–1974)	i	Education, household income, loneliness, marital status, employment, depression, smoker, alcohol use, physical activity, hypertension, diabetes, BMI, race	Coronary heart disease	Sex-disaggregation of multiple methods	No, women's SES gradient exceeds men's

## Results and discussion

### Gender differences in the SES gradient

In general, regardless of SES indicator or health outcome but with the important exception of heart disease, SES gradients were attenuated among women relative to men. This gender difference was consistent across the developed and developing world, measures of SES, and over time. Women of all SES levels tended to outlive men at the same level and the gap in health between lowest and highest socio-economic grade was narrower for women than men. At the same time, women tended to report greater morbidity, overall.

Indicators of SES varied from study to study and included combinations of individual or area education (10, 14–16, 18–20, 22, 23, 25, 26, 28, 30–32, 34, 36, 37, 41, 43, 44), individual or area income (14, 15, 23, 26, 29–31, 34, 37, 43, 44), occupational class and working conditions of self and/or spouse (11, 13, 15, 18, 20, 21, 23, 26, 28, 35–37, 39–44), housing quality (18, 20), subjective social position of women (19), household social position (35), access to economic resources (9), and neighborhood deprivation/inequality (12, 17, 18, 24, 33, 34, 38, 39, 41).

Health outcome measures for which men's SES gradients were shown to exceed those of women included mortality (10, 16, 17, 19, 20, 22–24, 26, 27, 33, 36, 38, 39), SRH (11, 13, 18, 28, 30, 37), and disability adjusted life years (21). Nevertheless, despite gender differences in the slope of the gradient, for both women and men, there was a clear and consistent association between increments in SES and in health.

When the health outcome was heart disease alone, and particularly when populations studied included those over age 65, a stronger SES gradient among men was not observed. Regardless of SES indicator or of whether the measure used was at the level of the individual, the community, or both, the SES gradient associated with cardiovascular disease appeared to be greater in women than men (12, 23, 36, 41, 43, 44). Deaths due to heart disease, therefore, amplified the SES gradient among women; however, overall gradients for mortality remained attenuated among women as compared to men.

### Gender and the limitations of specific measures of SES

The veracity of different measures of SES will vary between individuals and across groups such as women and men. As demonstrated in several of the papers reviewed, classifying women by individual occupational status could be problematic (13, 28, 35, 40). In a study of half of the states in the US, when SES was classified by individual occupation, ‘housewives’ were simply excluded (40). Lack of female engagement in the workforce could arise from extreme poverty or, alternatively, great wealth. Because of this, income of the man has traditionally been assumed to determine the socio-economic position of his family. However, in the most egalitarian countries it may be women, rather than their male partners, who earn a higher income and determine the SES of the family. Household income would seem to be a measure that corrects for this. However, inherent in it is an assumption of equal access to earned resources by both partners. In many cultures and countries, women have limited control over either their own or their spouse's income. Yet, another indicator of socio-economic position for all within a household might be occupational status of the partner with the higher grade if both are in the workforce.

And what about marital status, itself, as a predictor of health? Among a random sample of the Russian population, being married appeared to moderate the harm of deprivation or low SES (25). The interaction of marital status with education attainment may be different, however, for women and men. More highly educated women, but not men, tended to be single. The health benefits of education may, therefore, have been masked by the harms associated with being single and resulted in an apparent attenuation of the SES gradient associated with education/SES among women (20). Koskinen's evidence that controlling for marital status almost eliminated the gender gap in the association between education and mortality argues for being more inclusive and using multiple measures of SES and interactions between these, disaggregated by sex (20). One could speculate that having children might also interact with marital status in ways that have different health impacts for men and women. This theory was not tested, however, in any of the reviewed papers.

Control and satisfaction at work or home may also alter the SES gradient but in ways that differ for women and men. This gender difference was evident in some of the research reviewed. In a Spanish population, working conditions and job security seemed paramount as modifiers of the relationship between SES and health for men, while for women household material deprivation and domestic workload were key (11). A British study showed that measures of employment characteristics underestimated the SES gradient for women among whom social advantage of the household was a more accurate predictor of mortality (35). For men, findings were reversed. In Sweden, considered an egalitarian society, there were quite different gender effects. Domestic circumstances outweighed SES as determinants of health for men, while SES measures such as education or financial strain were of more significance among women (32).

This evidence points to gender differences in meanings of single measures and highlights the value of including multiple social indicators to limit gender bias. As each socio-economic measure is likely a proxy for other interconnected, but often unidentified, circumstances, the use of multiple indicators also acknowledges a probable web of causation rather than a single explanation for poorer health. As early as 1960, MacMahon discussed pushing beyond single and proximal risk factors to find explanatory frameworks, or the webs of causation in which isolated risks are embedded (45). To see this web, the parsimonious selection of explanatory variables favored in multivariate analyses must be balanced with adequate breadth of measures to avoid over-simplifying complex realities.

Harper and Strumpf conceptualized the web of causation with respect to the single indicator, education, as a predictor of health (46). They question whether the probability of illness for those with low education can decline to that of higher educated individuals by simply increasing years of education for the disadvantaged group, that is, by equalizing only one of many inequalities. Instead, they suggest that the variable, education, more likely summarizes and is a proxy for a complex network of life experiences, opportunities, and capabilities.

Kauffman expresses a similar construct with respect to income:

People with a given income value do not arrive at that value through a randomized process or anything remotely close to it; rather, they arrived at the observed value through a dynamic life trajectory that was shaped by an organized context of institutions and social relations. There can be no meaningful interpretation of an ‘independent’ effect in this setting …. (47)

In examining different outcomes for women and men, sex might be considered as a single explanatory factor. Studying the multiple traits that collectively define gender will give much deeper meaning than can sex differences alone and provide a web of causation.

Interconnections among social relations, life trajectories, economic circumstances, access to resources, exposures and responses to adversity, and not just chromosomes are among the realities that define and separate the experiences of men and women. When single socio-economic traits are considered to be independent predictors of health, evidence suggests that, relative to men, women are less susceptible to the SES gradient. However, as described above, SES characteristics are rarely independent, but instead, form a web, interacting with each other, as well as with other social circumstances such as connectedness, loneliness, risk taking (including, but not limited to smoking, alcohol abuse), or domestic equality. As an example from the reviewed papers illustrates, among British civil servants the modifying effect of characteristics such as social support, primary deprivation, and pessimism on the relationship of job insecurity to poorer health was markedly different for women and men (15). It would seem sensible to anticipate that the intersections of multiple social and environmental circumstances such as social isolation, spouse's SES, self and partner's education, or socio-economic position will not be similar for women and men and to search for such interactions.

### Research methods to identify gender differences

Including women and men in research on the relationship between SES and health is no guarantee that results will be reported separately for those most universal of categories. How, then, should gender be addressed? Studying interactions between sex and social circumstances such as education or income has been suggested as a method for identifying gendered effects (48). In a key paper among those reviewed, interactions between sex, living alone, and education level demonstrated gender differences in composite impact on health (20). This analytic approach avoided drawing erroneous conclusions about the associations of single social circumstances with the health outcomes being examined.

Sex disaggregation of findings was used in most of the reviewed studies to explore the gendered nature of the SES gradient. This brings us to the conundrum mentioned earlier. Embedded in the methodology of disaggregating data are assumptions about homogeneity and heterogeneity. If the underlying characteristics of all those surveyed are thought to arise independently and not to be related to some group characteristic, then participants are homogeneous and can be analyzed in aggregate. However, if, for example, membership in the group ‘men’ brings a common set of risks or traits that differ from the characteristics of those in the group ‘women,’ then this heterogeneity of the sample necessitates separate analyses for each group. Kaufman and MacLehose describe this further, with an example of how variation in relative risk estimates for women and men can arise from either individual or group differences:

Now it is necessary to make a binary decision between 2 opposing views of reality. The first possibility is that the 2 stratum-specific estimates are 2 independent draws from a single underlying sampling distribution of the homogeneous effect. The difference between these 2 values is therefore due to sampling variability alone. The second possibility is that the 2 stratum-specific estimates are each a draw from their own distinct stratum-specific distribution, because men and women do not share the same common underlying effect magnitude. In this case, the unstratified value is guaranteed to lie somewhere between the 2 stratum-specific estimates, but the specific value it takes will depend on the proportions of men and women in the study population. (49)

In only reviewing studies that reported SES gradient results for women and men separately, we will have preferentially identified papers that stratified data by sex (see Table 1). By either stratifying or examining interactions between sex and other independent variables, it becomes possible to identify whether there is heterogeneity, that is, a group effect that ‘pulls’ the data on the health impact of SES measures in different directions for women and men.

### Gender and choice of health outcome

Evidence from the reviewed papers showed a narrowed gap in SRH between most and least materially deprived women compared with men in many (13, 18, 28, 30, 37) but not all settings. In Sweden, a country with high gender equality, and in Britain, when the measures of SES included psychological strain and job satisfaction, women's gradient exceeded that of men (14, 15, 32). Failure to account for psychological suffering may explain some of the apparent blunting of women's SES gradient when self-reported health is the outcome. There is further evidence of this in the papers reviewed. For women, in particular, greater material deprivation can result in an emotional ‘wearing down’ not always captured by measuring physical morbidity or mortality. Ljung demonstrated that disability attributable to mental illness accounted for a larger proportion of poor health among women than men (21). When aspects of mental health (pessimism, psychological workplace circumstances, vigilance, etc.) were incorporated into the construct SRH, Ferrie found that women's SES gradient exceeded that of men (15). SRH is generally an outcome in which mental well-being is not specifically measured but may be embedded. Does the greater psychological strain from social circumstances often described by women affect their subjective reporting of health? Women tend to rate their health as poorer than do men of similar age, SES, and culture (37, 50). At the same time, women's SRH response across the SES gradient is attenuated relative to men's. These findings suggest group effects arising from some aspect of gender entwined in greater psychological wear and tear for women as a group. Including mental health as a component of overall self-reported health could more accurately approximate women's embodiment of social circumstances and might shift their SES gradient to approximate or even exceed that found among men.

Less readily explained, although perhaps also linked to gender differences in mental health is the paradox of lower and less variable female SRH across all SES grades alongside better physical health and greater longevity (51). Characteristics not clearly arising from current economic status may, never the less, have a central and gendered effect on SES and on health. Such characteristics include social isolation, pessimism, exposure to childhood adversity, marital status, or domestic situation (14, 15, 52). In the reviewed papers, including indicators of childhood adversity tended to equalize the SES gradient for women and men (29–31). Such evidence suggests once again that a broad view of psychosocial inputs will better explain the paradox of women's greater material deprivation, lower SRH overall, and apparent immunity to the SES gradient when only single and immediate economic measures of SES are considered.

Among the papers reviewed, only cardiovascular disease was associated with a greater SES gradient for women than men, and particularly when the age range studied extended beyond 65 (12, 16, 22, 36, 41–44). For example, a large study of mortality in France showed that the association between education or occupation and deaths overall, as well as specifically from cancer or injuries/violence, was greater for men than for women. However, women's SES gradient exceeded men's for death due to cardiovascular disease (36). Women's consistent disadvantage with respect to the leading cause of death in developed countries should increase the SES gradient for their mortality overall. On the other hand, breast cancer, another significant cause of death among women, does not follow the ‘wealth means health’ pattern. Its reverse SES gradient (greater wealth confers greater risk) offsets the cardiovascular disease effect and partially explained the observed SES – mortality blunting among women relative to men in several of the reviewed papers (3, 16, 40). These findings raise the question of whether observed variations in the SES gradient between men and women are explained by gender differences in causes of death. Koskinen and Martelin (20) attempted to answer this by controlling the cause of death in a study of income differences in mortality in Finland. The authors created an ‘imaginary situation’ where mortality for each sex remained unchanged by age but women's causes of death were redistributed to follow those of men. This method narrowed, but did not eliminate, gender differences in the SES gradient. Their results imply that women's smaller socio-economic inequality in mortality is partially, although not completely, explained by gender differences in causes of death.

### Ecological and multilevel measures: limiting gender bias

Although compositional effects (clustering of poor people) partially account for the lower health status of materially deprived neighborhoods, there may be consequences of living in such areas that have an impact on all residents. How area characteristics actually affect the health of those living in an area is unknown. Similarly, the effect of context, that is, of living in a particular environment, is somehow entangled in area social norms, environmental conditions, access to resources, safety, and other neighborhood qualities that extend beyond compositional measures of critical mass (38, 53). For example, does a high level of area unemployment harm only because a large number of residents suffer the individual health harms of unemployment or are there effects such as hopelessness that cast a health shadow over all residents regardless of employment status? Furthermore, could that neighborhood despondency have a differential impact on women and men?

In the studies reviewed, and in keeping with findings reported elsewhere when compositional indicators were measured their impact on health was real, although generally weaker than that of individual circumstances. Area material deprivation tended to have more impact on the health of men than women (18, 19, 24, 33, 38, 39, 54). In the two papers that measured area social capital, however, the SES gradient for women exceeded that found among men in one (14), but not the other study (19). The finding of greater harm from area deprivation for the health of men versus women seems perplexing because 1) women's well-being is generally more aligned with neighborhood social characteristics than is that of men and 2) the sphere of influence on women's health tends to be home and immediate environment, whereas for men it is the workplace (11, 55). Perhaps, however, compositional indicators of area deprivation hide women's real susceptibility to area effects. The gender biases inherent in individual level indicators described earlier will be magnified in aggregating these up to neighborhood level and will skew evidence. As a number of the reviewed studies showed, individual education can be a poor predictor of health for women. What meaning can then be derived from aggregating this ambiguous individual measure to an area-level proportion, as did many of the ecological and multilevel studies reviewed (12, 18, 19, 24, 33, 41)?

Even more problematic is the use of area deprivation indices to capture multiple measures of SES in a single variable such as the Townsend or Carstairs indices (5, 6). If the individual-level indicators of SES that are aggregated up to compositional variables differ in meaning for women and men, collapsing these even further into single indices will compound ambiguities and biases. Inherent in any index of deprivation is an assumption that the relative impact of each component of the index on individual health will be stable and equal across the populations and settings studied. It might be, however, that the equal weighting of components of an index does not reflect gendered realities. For example, in finding a greater impact of area deprivation on American men than women, Singh used an index that included ‘percentage of single parent households’ (38). One could hypothesize that for women, and particularly for single mothers living in an area where this proportion is high, might augment a sense of belonging, comfort, safety, and connectedness. This could then be an indicator with inverse meaning for women and men, one that predicts health harm for men but not for women. As a result, an index that includes ‘percentage of single parents’ may obscure rather than characterize area deprivation. Other components of indices could be subjected to similar scrutiny with respect to differential meanings for women and men.

There is evidence (56) that in the US, the single indicator, percentage of persons below poverty level, is as robust a measure of area SES as are the Carstairs or Townsend deprivation indices (5, 6). This may speak as much to bias arising from unidentified interactions among components of indices with gender, as to the strength of Krieger's single measure, or, put another way, to the information that is lost when only average compositional measures form a summary index.

Although infrequently used, there are a number of contextual, rather than compositional indicators of area SES and gender equality that lack the intrinsic bias of aggregated individual characteristics (57). Some measures of contextual neighborhood status, not derived from and having no individual level correlate, are less likely to diverge by gender. Instead of using the proportion of an area's population with low income to indicate area SES, measures of ratios of female to male income or the percentage of both impoverished women and men were calculated in several of the reviewed papers (17, 19). Using separated proportions of women and of men may avoid the inevitable errors associated with sex-aggregated measures. Other gender-sensitive area indicators might include ratios of female to male years of education, rates of childbearing under age 18, or the proportion of women in public office. Each of these measures how gender equality interacts with neighborhood SES. Kawachi examined the impact of area gender equality on the relationship between SES and health by measuring women's political participation, employment and earnings, economic autonomy, and reproductive rights (17). Gender equality appeared to be a strong predictor of longevity for all residents. The association remained with adjustment for individual SES factors suggesting that limited opportunities for women affected the pathway from income to poor health for men and women. Similarly, in the US greater equality for women improved everyone's health behaviors (34). However, in Sweden, higher neighborhood gender equality gave rise to poorer health for all (9). Overall, the explanatory values of contextual SES and of gender equality are strong enough to recommend their inclusion in area or multilevel studies of the SES gradient.

### The gendered effect of social capital

Individual, ecological, and multilevel analyses of social determinants of health sometimes differentiate between material and social deprivation. Including measures of, for example, social capital as well as material circumstances and considering differences between men and women in the interactive and additive effects of these may be of explanatory value (56). One reviewed paper demonstrated that neighborhoods with higher social capital had a positive impact on the SRH of women but not on men (14). Similarly, in a study on predictors of heart disease for men and women, a lack of social capital (measured as loneliness) was associated with increased incidence of heart disease among women but not among men (44). The gendered effect of social capital was not consistent across all studies. Among UK civil servants, the lack of social support predicted all causes and cardiovascular mortality for men but not for women (42). However, in this same study, women with higher SES reported lower social support, whereas men did not. Perhaps, the overall blunting of the SES gradient among these women reflected the competing benefit of higher SES and harm of lower social support. Further research on the interconnections between social capital, gender, and SES and their impact on health is needed to differentiate pathways, modifiers, and confounders.

### Limitations

Finding research on gender, SES gradients, and health is hampered by the lack of MeSH terms such as gender, and ambiguity of terminology around sex differences. We searched titles, abstracts, and keywords as well as MeSH terms but acknowledge that relevant papers for this analysis will have been missed. Of necessity, we considered only those papers with findings differentiated by sex. Had more data been sex-disaggregated more information could have been included and might have changed our interpretations. The solution to this potential bias, and one of our recommendations, would be sex stratification of all SES gradient research.

Assessing heterogeneity of a cohort, overall, without assuming homogeneity within the groups ‘men’ and ‘women’ should be embedded in research methodology. The categories of ‘women’ and ‘men’ are standard subgroups for analyses as they are universal. At the same time, we recognize that their use may construct a problematic dichotomy where everyone is seen as either male or female, reinforcing stereotypic ideas and neglecting that masculinity and femininity have multiple meanings both within and across cultures, regions, and countries. Research methods that address both ‘within’ and ‘between’ group variation will lessen stereotyping intrinsic to simplistic dichotomizing by sex and acknowledge diversities and commonalities within groups. Including a range of social traits as, and interactions of, independent variables enables researchers to move beyond assumptions of homogeneity within groupings and toward an understanding of the characteristics that unify and differentiate those within each category.

Finally, can connections between gender equality and the SES gradient on health for men and women be studied in settings of apparent universal equality? As Geoffrey Rose described in his classic paper, Sick Individuals and Sick Populations, standard cohort studies typically used to assess the SES gradient cannot reveal the impact of ubiquitous risks, exposures, or characteristics on health (58). Quantifying the association between characteristics and health outcomes requires variability in those characteristics to calculate a relative rate of harm or benefit arising. If, for example, everyone being studied has a high education, then the relative contribution of this trait to health could not be determined. If a country had universal gender equality, it would be difficult to measure within country contributions of, or modifying by this contextual characteristic. However, recent measures of neighborhood gender inequality demonstrate that in one relatively egalitarian country, Canada, variability in these measures is of enough magnitude to enable comparisons across areas (59).

## Conclusion

Researchers will rarely uncover unimagined outcomes. Failure to consider that influences of the lived environment on morbidity and mortality may differ for women and men demonstrates a lack of imagination rather than of effect. Using sex-aggregated data embeds into methodology the erroneous assumption that there are no group effects related to gender and blinds researchers to gender differences. We would suggest consistent use of sex-disaggregated data as the studies reviewed indicated that differences in the socio-economic gradient between women and men are the norm rather than the exception.

Some of the observed variability between men and women may, however, be an artifact of bias in methodology. Errors can arise from assuming independence of ‘independent’ indicators of social and economic circumstances such as income, marital status, or social connection. Because these variables, in reality, form an interdependent web of causation and because interdependence is different for women and men, associations between outcomes and single social characteristics will often be misleading. Ignoring, for example, gender differences in interactions between marital status and education among women would have biased findings in the single study to address this (20). Considering gender differences increased the steepness of the SES gradient's impact on health among women to more closely approximate that of men. We would, therefore, further suggest including characteristics such as domestic circumstances, social connectedness, or childhood adversity when studying the SES–health relationship to more accurately measure and better understand gender differences in findings. SES is a summative term for many unmeasured social circumstances. Including a variety of these, and particularly identifying social characteristics whose modifying or interactive effect is different for women and men, will help explain the apparent paradox of women's relative immunity to the harms of their greater poverty.

Even single indicators of SES or of self-rated, physical or mental health can have gendered meanings that, if ignored, may bias research. For men, material deprivation seems to be associated with physical health, whereas for women the impact may be more on mental health. We recommend using outcome measures that incorporate mental as well as physical health to better address the full scope of well-being, particularly for women. When SRH is the outcome, the blunted SES gradient observed among women may arise from gender differences in the measure itself, rather than from the impact of socio-economic circumstances on it. Across time and place, women's ratings of their own health are lower and more resistant to the benefits of prosperity than those of men. Explanations for gender differences in SRH seem anomalous, given women's relative longevity. It is, therefore, worth addressing possible inherent differences in how women and men define SRH, and how the differences may skew findings making it seem like the inputs (the indicators of social and economic status), rather than the chosen output (SRH) explain women's apparent protection from the health impact of SES inequalities.

Differences in causes of death for women and men may blunt the apparent SES gradient among women. In developed countries, cardiovascular disease is generally the leading cause of mortality for all, and one with a steeper SES gradient among women. However, the reverse SES gradient for breast cancer, with wealth conferring greater risk, flattens the slope of women's SES gradient for mortality, overall. Adjusting for sex-specific causes of death would untangle SES gradient effects on mortality from sex differences in causes of mortality.

Compositional area indicators of SES that are derived by aggregating individual measures can compound inherent gender biases. We suggest using indicators such as ‘proportion of women’ and, as a separate measure, ‘proportion of men’ with a particular SES characteristic such as ‘living below the poverty line’, and contextual indicators of area SES rather than sex-aggregated proportions. Evidence of associations between area gender equality and health suggest the merit of consistently using such indicators in ecological or multi-level SES-health research. The protective effect, usually but not always more pronounced in women, of individual or area social capital makes this another measure we recommend including.

Socio-economic circumstances have a real and sustained impact on individual health. The SES gradient appears stronger for men than women for all health outcomes other than heart disease. However, moving beyond assessment of individual risk factors to consider webs of causation can explain much of the observed gender difference in the socio-economic gradient and will deepen the understanding of the pathways from SES to morbidity and mortality.
